# Artificial intelligence for diagnosis of mild–moderate COVID-19 using haematological markers

**DOI:** 10.1080/07853890.2023.2233541

**Published:** 2023-07-12

**Authors:** Krishnaraj Chadaga, Srikanth Prabhu, Vivekananda Bhat, Niranjana Sampathila, Shashikiran Umakanth, Rajagopala Chadaga

**Affiliations:** a Department of Computer Science and Engineering, Manipal Institute of Technology, Manipal Academy of Higher Education, Manipal, India; b Department of Biomedical Engineering, Manipal Institute of Technology, Manipal Academy of Higher Education, Manipal, India; c Department of Medicine, Dr. TMA Hospital, Manipal Academy of Higher Education, Manipal, India; d Department of Mechanical and Industrial Engineering, Manipal Institute of Technology, Manipal Academy of Higher Education, Manipal, India

**Keywords:** Artificial intelligence, clinical markers, COVID-19, decision support system, machine learning

## Abstract

**Objective:**

The persistent spread of SARS-CoV-2 makes diagnosis challenging because COVID-19 symptoms are hard to differentiate from those of other respiratory illnesses. The reverse transcription-polymerase chain reaction test is the current golden standard for diagnosing various respiratory diseases, including COVID-19. However, this standard diagnostic method is prone to erroneous and false negative results (10% -15%). Therefore, finding an alternative technique to validate the RT-PCR test is paramount. Artificial intelligence (AI) and machine learning (ML) applications are extensively used in medical research. Hence, this study focused on developing a decision support system using AI to diagnose mild-moderate COVID-19 from other similar diseases using demographic and clinical markers. Severe COVID-19 cases were not considered in this study since fatality rates have dropped considerably after introducing COVID-19 vaccines.

**Methods:**

A custom stacked ensemble model consisting of various heterogeneous algorithms has been utilized for prediction. Four deep learning algorithms have also been tested and compared, such as one-dimensional convolutional neural networks, long short-term memory networks, deep neural networks and Residual Multi-Layer Perceptron. Five explainers, namely, Shapley Additive Values, Eli5, QLattice, Anchor and Local Interpretable Model-agnostic Explanations, have been utilized to interpret the predictions made by the classifiers.

**Results:**

After using Pearson’s correlation and particle swarm optimization feature selection, the final stack obtained a maximum accuracy of 89%. The most important markers which were useful in COVID-19 diagnosis are Eosinophil, Albumin, T. Bilirubin, ALP, ALT, AST, HbA1c and TWBC.

**Conclusion:**

The promising results suggest using this decision support system to diagnose COVID-19 from other similar respiratory illnesses.

## 1. Introduction

The SARS-CoV-2 pandemic has affected all facets of life, including healthcare, education, the environment and the economy [1]. It has also raised concerns about how medical facilities and healthcare systems can respond to a new virus. As of 18 April 2023, 762,791,152 cases were reported, including six million deaths [2]. Eventually, vaccines such as BioNTech, Sinopharm, Moderna and Covaxin were developed to combat COVID-19 [3]. These vaccines were distributed to people all around the world. The vaccines effectively prevent a severe COVID-19 prognosis in most people [4].

**Figure 1.**
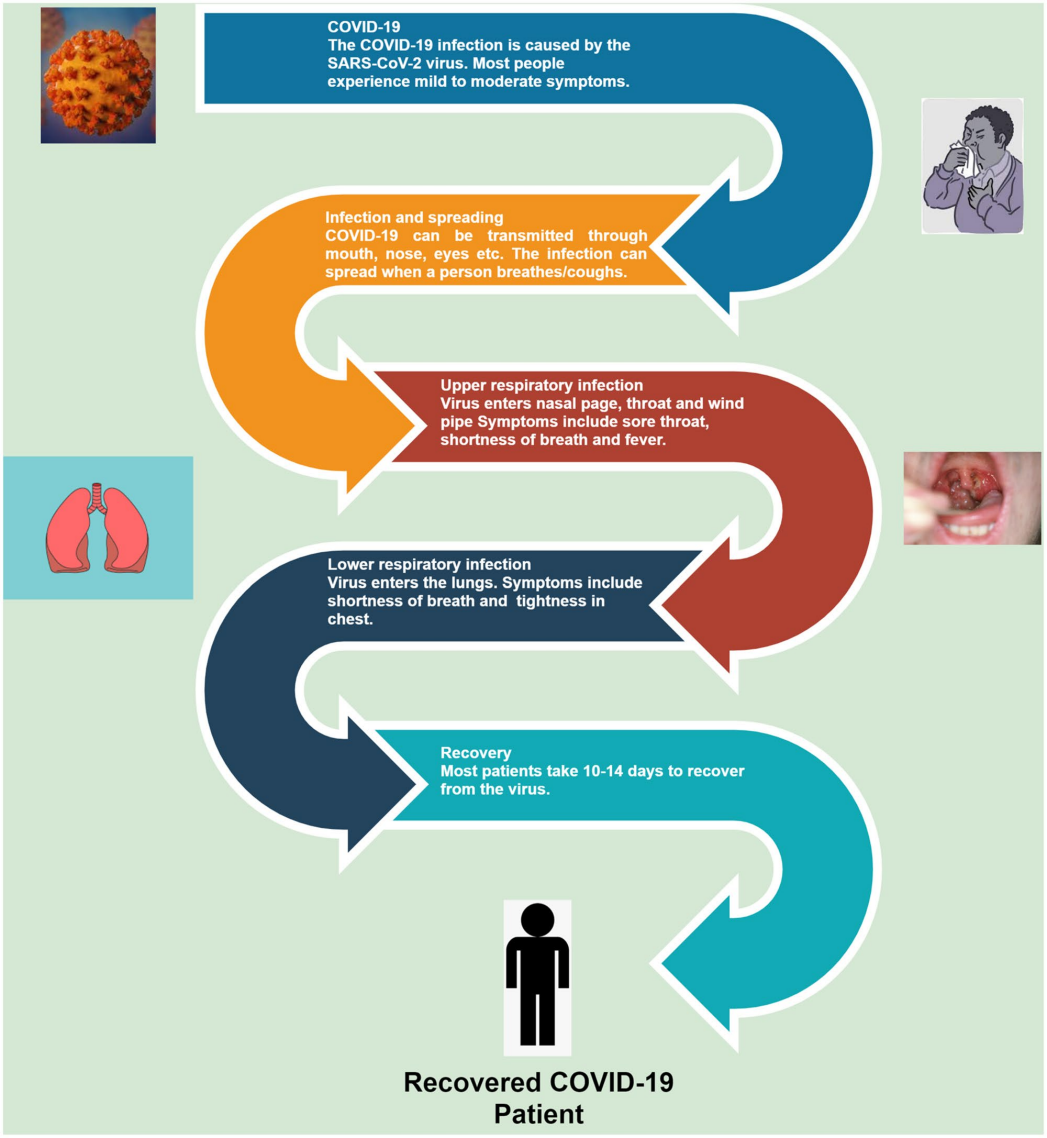
COVID-19 transmission and recovery.

The lifecycle of COVID-19 is described in Figure 1. Due to the wide range of clinical manifestations that can occur in patients, the ongoing worldwide spread of COVID-19 has made diagnosis difficult for clinicians. Seasonal influenza has made diagnosis more challenging because influenza and COVID-19 cause similar symptoms [5]. Given that both viruses co-exist, it is crucial to differentiate them to manage patients at an elevated risk of infection, some of which may be virus-specific. COVID-19 is detected using a standard test called the reverse transcriptase polymerase chain reaction (RT-PCR). However, it consumes a lot of time to generate results. False negative and misclassified results have also been observed. It also requires special equipment and reagents that might only be available in some hospitals, especially in underdeveloped countries [6]. Hence, an alternative method to diagnose COVID-19 is required. Several methods, including computed tomography (CT) images, ultrasound, X-rays, voice-based analysis, clinical markers and magnetic resonance imaging (MRI), have been used for COVID-19 diagnosis [7].

Machine learning (ML) and deep learning (DL) applications are being heavily used in healthcare settings for diagnosis, screening, prognosis and risk assessment [8]. The models can enhance a physician’s decision-making process using computational and visualization methodologies. The advancements listed above are mainly due to the emergence of relevant medical datasets and algorithms [9]. The interpretability and comprehensibility of modern artificial intelligence (AI) applications should also be examined before deploying them in actual scenarios. A model’s rationale behind a prediction must be clear for doctors to trust the diagnosis. This demonstrates why most ML models are just used as prototypes in the medical field. Some medical professionals hesitate to use AI applications that are hard to trust and understand [10]. Explainable AI(XAI) tries to interpret the predictions using model estimators, global/local explanations and rule-based estimations. Further, the explainers use visualization techniques, making it easier for physicians to understand the predictions [11].

White blood cells, basophils, monocytes, neutrophils, lymphocytes, eosinophils, aspartate aminotransferase (AST), alanine transferase (ALT), albumin and other clinical markers have proven to be important for COVID-19 diagnosis [12]. This presents a rare opportunity for researchers to simultaneously explore features which influence patient diagnosis and develop cutting-edge testing methodologies for COVID-19 diagnosis. A few studies that use ML in the battle against COVID-19 are discussed below.

Gavriilaki et al. [13] used a rigorous algorithm to justify severe COVID-19 in patients. Clinical data of 97 patients were considered for this research. Asteris et al. [14] used neural networks to predict mortality in severe COVID-19 patients. One hundred and thirty-three patients were considered for this research. According to the study, the most markers are THBD and C3a levels. In another research, fuzzy ensemble models and transfer learning were used to detect COVID-19 using chest X-rays [15]. Two thousand three hundred and thirteen images that consisted of COVID-19, other pneumonia and normal patients were considered. A maximum accuracy of 99.5% was obtained. In another research, ML models were used to predict COVID-19 cases [16]. Three countries were considered that included United Kingdom, Italy and Australia. Asteris et al. [17] used mathematical modelling to understand the risks of COVID-19. The number of COVID-19 deaths in six different countries was analysed. Five laboratory markers were used to predict COVID-19 severity in another research [18]. Two hundred and forty-eight patients and 25 clinical markers were considered. A maximum accuracy of 96% was achieved by the ANN model.

COVID-19 mortality was predicted using ML in yet another research [19]. A Mexican dataset was considered and a maximum accuracy of 96% was obtained. COVID-19 was diagnosed using routine blood tests in another research [20]. Four classifiers were used for prediction and the random forest obtained an accuracy of 92%. This study uses ML and DL algorithms to develop an early decision support model that can diagnose COVID-19 from other similar respiratory illnesses. The other contributions of this research are given below:

- The mild–moderate COVID-19 and non-COVID-19 influenza-like illness (ILI) patient data have been collected from two Indian hospitals. Ethical clearance was obtained prior to data collection.
- Fifteen feature selection algorithms have been compared to understand the most important markers.
- A custom multi-level stacked ML architecture has been developed for diagnostic prediction. The results have been further compared with DL algorithms, including deep neural networks (DNN), long short-term memory networks (LSTM), one-dimensional convolution neural networks (1D-CNN) and residual multi-layer perceptron (ResMLP).
- XAI methods such as Shapley additive values (SHAP), QLattice, local interpretable model-agnostic explanations (LIME), Eli5 and Anchor are utilized to understand the model predictions. Explainers such as QLattice, Eli5 and Anchor have been rarely used in medical research.
- The results obtained in this study have been compared with relevant medical literature.

The study continues as follows: Materials and methods are covered in Section 2. In-depth explanations of feature selection, data description, pre-processing and ML approaches are provided in this section. Results and discussions are presented in Section 3. This section describes the outcomes of the ML and DL models, their interpretation and future discussions. The final section contains the conclusion and recommendations for further study.

## 2. Materials and methods

### 2.1. Description of the dataset

The COVID-19 and non-COVID-19 ILI patient datasets were obtained from Dr TMA Pai Hospital and Kasturba Medical College. The above hospitals are situated in the Udupi District, India. Ethical clearance was obtained to carry out this study (ethical clearance ID: IEC: 613/2021). Informed verbal consent was also taken from the patients before conducting this study. The details of patients who underwent the RT-PCR test between April 2022 and December 2022 were considered. Blood test results were obtained from 870 patients (270 non-COVID-19 ILI, 300 mild COVID-19 and 299 moderate COVID-19 patients). Severe COVID-19 patients were not considered in this study since the number of severe cases has drastically reduced after introducing COVID-19 vaccines. Further, these patients’ markers vary drastically, making the classification biased towards severe COVID-19 patients. Every patient was asymptomatic or had mild–moderate influenza symptoms such as myalgia, cough and fever. A clear description of the parameters is made in Table 1.

**Table 1.** Dataset description.

No.	Feature name	Description (units)	No.	Feature name	Description (units)
1	Age	Age of a patient in years. Only patients above 18 years of age were included in this study. (in years)	14	Haematocrit	It measures the proportion of red blood cells in the blood (in %)
2	Haemoglobin	A protein that transports oxygen to all the organs in the body. A decrease in haemoglobin count causes anaemia (g/dL)	15	Neutrophil	A type of white blood cell that helps in fighting diseases. An increase in neutrophil count is observed in COVID-19 patients (in %)
3	Total white blood cells (TWBC)	Total number of white blood cells in the human body. They combat infections and are a component of the immune system (10^3^/μL)	16	Neutrophil to lymphocyte ratio (NLR)	The number of neutrophils to the number of lymphocytes. NLR levels are known to elevate in COVID-19 patients.
4	Lymphocyte	A type of white blood cell. They are produced in the bone marrow. A decrease in lymphocyte count has been observed in many COVID-19 patients (in %)	17	Eosinophil	A type of white blood cell. High/low eosinophil count can be dangerous if not treated (in %)
5	Monocyte	A type of white blood cell. Monocyte count is known to elevate in COVID-19 patients (in %)	18	Basophil	It is a type of white blood cell. It helps the body in fighting infections (in %)
6	Platelet	Platelets stop bleeding by forming blood clots (in%)	19	Creatinine	Excess creatinine levels indicate damage to the kidneys (mg/dL)
7	Urea	Elevated urea levels can damage the kidneys (mg/dL)	20	Potassium	Electrolytes that keep a balance between fluids and blood volume (mmol/L)
8	Sodium	Electrolytes that keep a balance between fluids and blood volume. Excess sodium can cause hypertension (mmol/L)	21	Direct bilirubin (D. bilirubin)	Bilirubin is converted in the liver into a substance that can be removed from the body. This is called D. bilirubin (mg/dL)
9	Total bilirubin (T. bilirubin)	Direct bilirubin and indirect bilirubin combine to form T. bilirubin (mg/dL)	22	Alanine transaminase (ALT)	It is a liver enzyme, which is mainly present in the liver itself (IU/L)
10	Aspartate transaminase (AST)	It is a liver enzyme mainly present in the heart, kidneys, lungs and brain (IU/L)	23	Protein	Total protein content present in the blood (g/dL)
11	Alkaline phosphatase (ALP)	It is a liver enzyme mainly present in the bones and liver (IU/L)	24	HbA1c	It indicates how much glucose is present in the haemoglobin. Elevated levels of HbA1c can lead to diabetes (in %)
12	Albumin	It is a protein generated by the liver. It prevents fluid from seeping into the blood tissues (g/dL)	25	Label	RT-PCR test results. Positive result indicates a COVID-19 patient. Negative results indicate a non-COVID-19 patient.
13	Gender	Sex of a patient (male/female)			

### 2.2. Dataset preprocessing and feature selection

Data preprocessing involves various steps such as removing null values, variable encoding, data standardization, removing outliers and data balancing. In this study, 23 continuous and two categorical attributes (Gender and Label) are present. Generally, missing values are replaced using mean, median and other imputation methods. We used the median to replace the continuous attributes since outliers do not affect them. The categorical variable ‘Gender’ had no missing values. In this research, ‘JAMOVI’ was utilized to perform descriptive statistical analysis [21]. Researchers use it as an open-source statistical tool to perform extensive statistical analysis. Table 2 describes the several statistical measures such as mean, median, standard deviation, interquartile range and percentiles.

**Table 2.** Descriptive statistical analysis COVID-19 dataset (continuous variables).

	ILI	*N*	Attribute mean	Attribute median	Standard deviation	Inter quartile range	25th percentile	50th percentile	75th percentile
Age	Non-COVID-19 ILI	270	52.784	54	19.93	32	36	54	68
	COVID-19	599	52.306	54	18.664	29	38	54	67
Hb (haemoglobin)	Non-COVID-19 ILI	270	12.305	12.4	1.824	1.675	11.6	12.4	13.275
	COVID-19	599	13.065	12.9	4.335	2.45	11.9	12.9	14.35
PCV% (haematocrit)	Non-COVID-19 ILI	270	36.406	36.5	5.133	4.675	34.2	36.5	38.875
	COVID-19	599	39.263	38.5	18.32	6.5	35.55	38.5	42.05
TWBC	Non-COVID-19 ILI	270	8.497	7.95	4.316	2.425	6.55	7.95	8.975
	COVID-19	599	6.429	5.7	3.501	3	4.5	5.7	7.5
Neutrophil	Non-COVID-19 ILI	270	66.599	67.2	12.854	14.525	60.05	67.2	74.575
	COVID-19	599	67.457	68.3	12.725	16	60	68.3	76
Lymphocyte	Non-COVID-19 ILI	270	22.342	21.8	11.159	13.625	15	21.8	28.625
	COVID-19	599	22.105	20.9	11.171	14	14	20.9	28
NLR	Non-COVID-19 ILI	270	4.615	3	5.4	2.75	2	3	4.75
	COVID-19	599	4.431	3	9.404	3	2	3	5
Monocyte	Non-COVID-19 ILI	270	8.223	7.8	3.218	3.075	6.625	7.8	9.7
	COVID-19	599	9.06	8.7	5.816	4.7	6.3	8.7	11
Eosinophil	Non-COVID-19 ILI	270	1.99	1.5	1.904	1.725	0.8	1.5	2.525
	COVID-19	599	0.849	0.3	1.544	1	0	0.3	1
Platelet	Non-COVID-19 ILI	270	266.642	263.5	111.76	107.75	215	263.5	322.75
	COVID-19	599	238.314	224	93.212	103.5	180	224	283.5
Basophil	Non-COVID-19 ILI	270	0.492	0.4	0.423	0.3	0.3	0.4	0.6
	COVID-19	599	0.492	0.3	3.756	0.2	0.2	0.3	0.4
Urea	Non-COVID-19 ILI	270	28.974	21.5	38.3	5.75	19.25	21.5	25
	COVID-19	599	25.608	22	15.313	13	17	22	30
Creatinine	Non-COVID-19 ILI	270	0.938	0.8	0.681	0.2	0.7	0.8	0.9
	COVID-19	599	0.912	0.8	0.4	0.3	0.7	0.8	1
Sodium	Non-COVID-19 ILI	270	133.911	135	5.163	3.75	132.25	135	136
	COVID-19	599	135.94	137	7.327	5	134	137	139
Potassium	Non-COVID-19 ILI	270	4.126	4.1	0.387	0.3	4	4.1	4.3
	COVID-19	599	4.257	4.1	2.338	0.6	3.8	4.1	4.4
T. bilirubin	Non-COVID-19 ILI	270	0.716	0.5	1.127	0	0.5	0.5	0.5
	COVID-19	599	0.538	0.4	0.533	0.3	0.3	0.4	0.6
D. bilirubin	Non-COVID-19 ILI	270	0.362	0.2	0.899	0	0.2	0.2	0.2
	COVID-19	599	0.229	0.2	0.278	0.2	0.1	0.2	0.3
AST	Non-COVID-19 ILI	270	46.719	33	62.99	0	33	33	33
	COVID-19	599	42.891	33	47.299	24	24	33	48
ALT	Non-COVID-19 ILI	270	41.648	35	34.909	2.375	33.375	35	35.75
	COVID-19	599	38.364	28	39.985	27	18	28	45
ALP	Non-COVID-19 ILI	270	95.622	89	44.133	0	89	89	89
	COVID-19	599	83.2	76	45.98	30	63	76	93
Protein	Non-COVID-19 ILI	270	7.021	7	0.414	0	7	7	7
	COVID-19	599	7.075	7.2	0.512	0.2	7	7.2	7.2
Albumin	Non-COVID-19 ILI	270	3.847	3.9	0.349	0	3.9	3.9	3.9
	COVID-19	599	4.081	4.1	0.458	0.4	3.9	4.1	4.3
HbA1c	Non-COVID-19 ILI	270	6.1	5.8	1.311	0	5.8	5.8	5.8
	COVID-19	599	6.54	5.9	1.728	1.8	5.4	5.9	7.2

**Figure 2.**
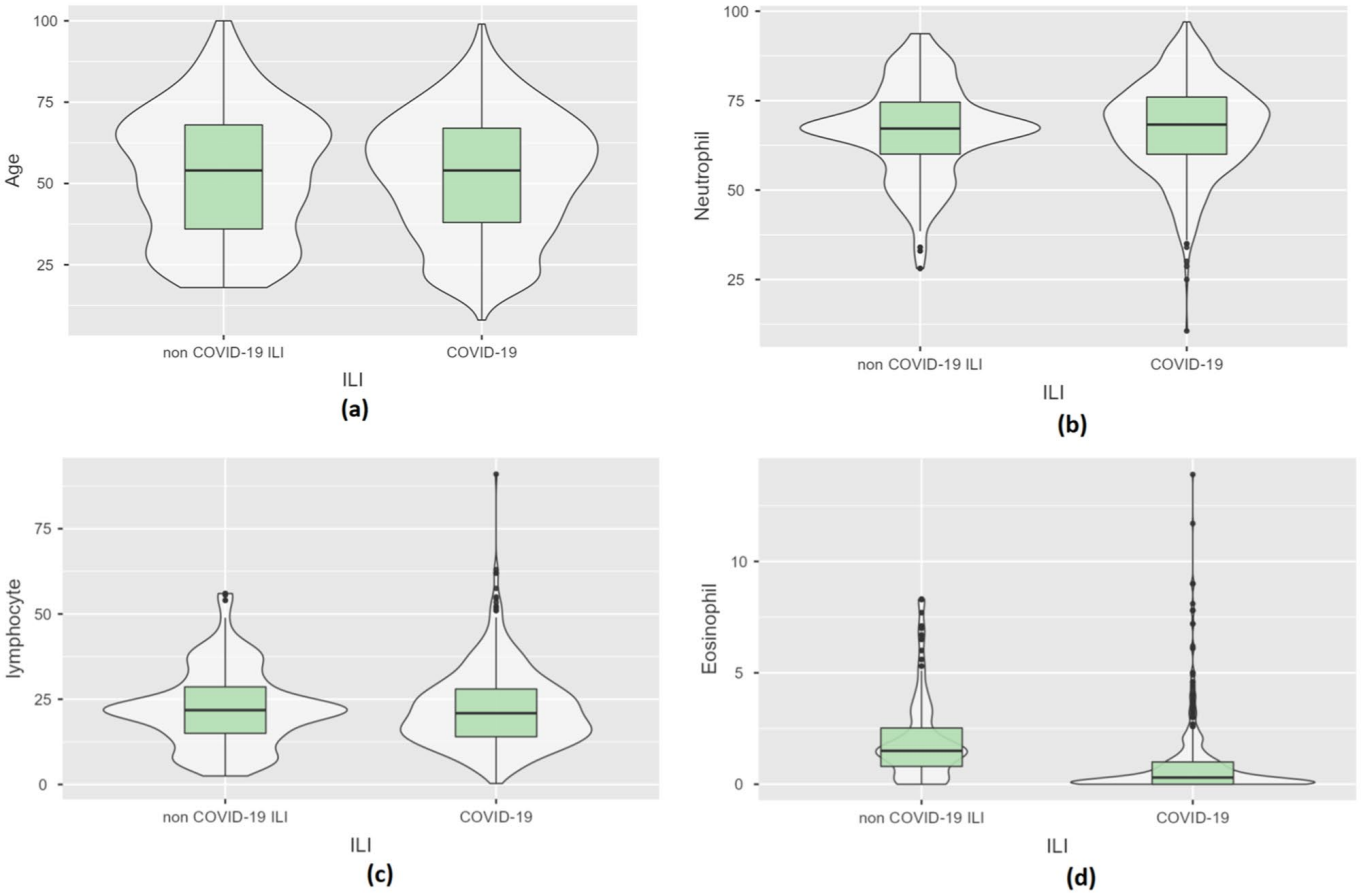
Box plots for four features. (a) Patient age, (b) neutrophil count, (c) lymphocyte count and (d) eosinophil count.

**Figure 3.**
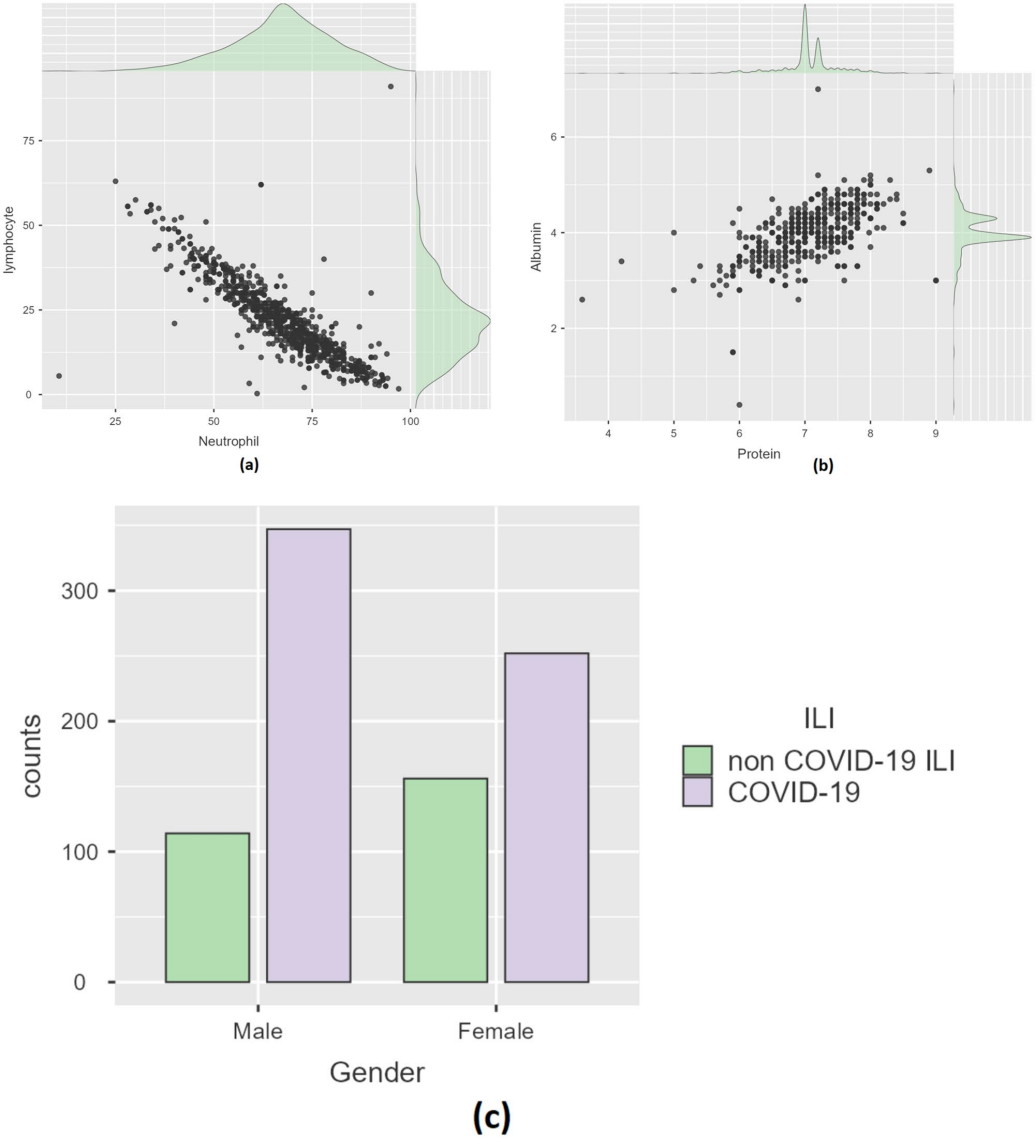
(a) Scatter plot for neutrophil and lymphocyte, (b) scatter plot for albumin and protein and (c) bar graph for gender representation.

For better data visualization, bar graphs, scatter plots and violin plots were generated, as shown in Figures 2 and 3. From Figure 2, it can be seen that there was not much difference in age between COVID-19 and non-COVID-19 patients. From the figure, it can also be seen that eosinophil levels were slightly elevated in non-COVID-19 patients. Neutrophil levels were slightly elevated, and lymphocyte levels were slightly lower in COVID-19 patients. Further, many attributes had outliers. We did not handle outliers in this study since it would make the models more biased during testing.

From Figure 3(a), it can be seen that neutrophils and lymphocytes are inversely proportional. A linear relationship exists between the two. From Figure 3(b), it can be seen that protein and albumin are directly proportional to each other. A linear relationship also exists between the above two attributes. The gender composition of the dataset is described in Figure 3(c). The non-COVID-19 cohort consisted of 114 male and 156 female patients. Three hundred and forty-seven male patients and 252 female patients were present in the COVID-19 cohort. One-hot encoding was performed on the ‘Gender’ attribute [22]. Before developing a model, categorical attributes must be converted into integers since many classifiers cannot handle string values. After data pre-processing, the dataset was split into training and testing (80:20). Data scaling is essential in ML. When there is a substantial difference between data points, the effectiveness of the models is negatively impacted. Furthermore, the algorithms favour attributes with greater values, regardless of the metrics utilized. This study used standardization to scale the data [23]. In standardization, the standard deviation of the attribute is assigned as one and the data points are grouped around the features’ mean.

Medical data are frequently unbalanced, which distorts the proportion of the data. From Table 2, it can be seen that COVID-19 cases are almost double that of non-COVID-19 ILI cases. The classifiers favour the category with more occurrences. Hence, data balancing is essential. This study used the Borderline-SMOTE method to balance the training data [24]. However, the testing data were not subjected to balancing to protect the integrity of the data.

**Figure 4.**
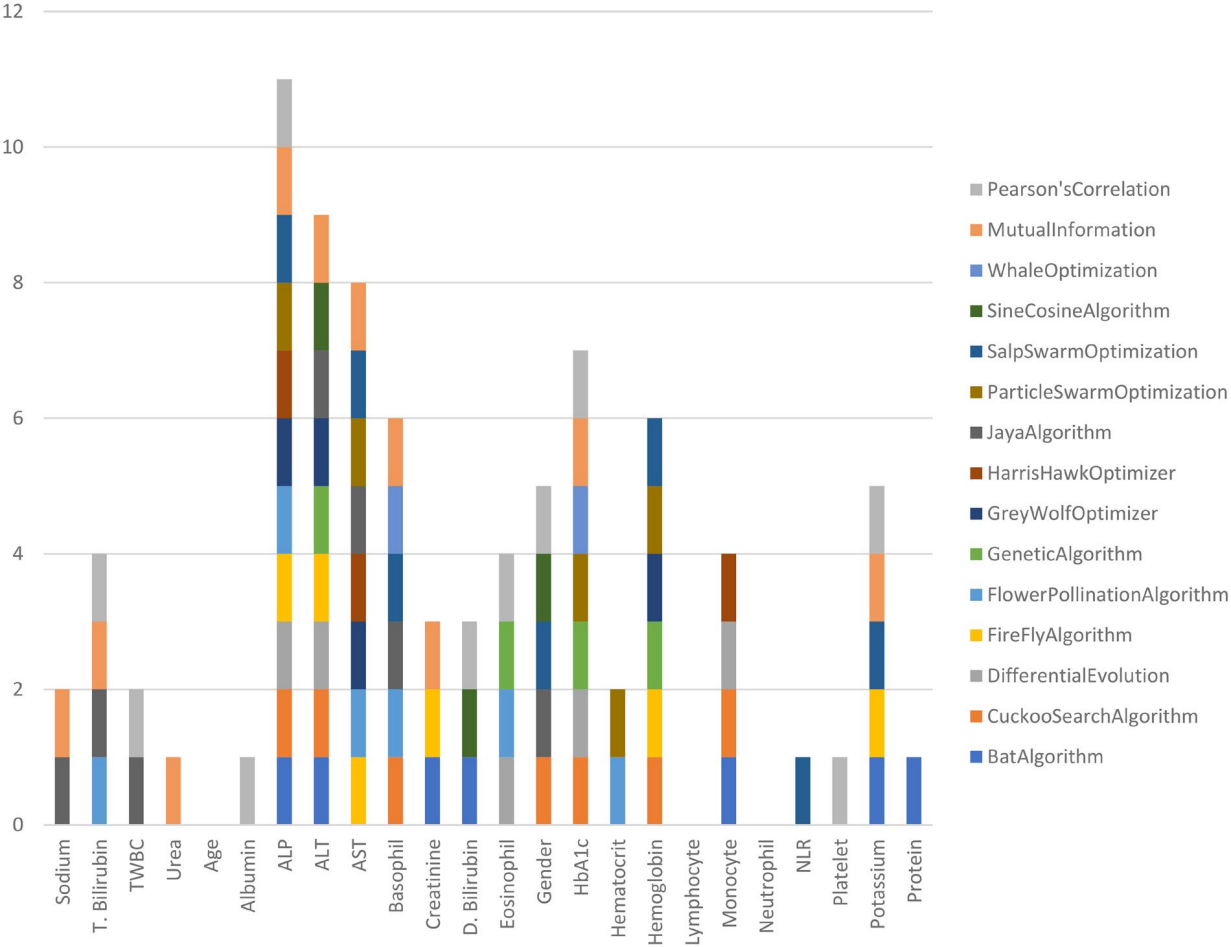
Features chosen by various algorithms.

Feature selection is utilized for choosing the essential attributes. Due to the widespread adaption of modern technologies and intelligent systems, massive amounts of data have been produced. Concerns such as noise and duplication are considerably reduced once feature selection is completed [25]. Fifteen feature selection methods have been compared in this study. Table 3 describes the feature selection methods, the number of attributes chosen by each technique and the names of the features chosen. The table shows that the Harris Hawks optimization, whale optimization and sine cosine algorithm chose the least number of features. Most algorithms choose between 5 and 10 features. Figure 4 gives a pictorial description of the features chosen by various algorithms. From the figure, it can be observed that the most important parameter chosen is ALP. Many methods also chose liver enzymes such as ALT and AST. A few algorithms also considered HbA1c, haemoglobin, monocytes and potassium. The techniques did not consider three attributes: age, neutrophil and lymphocyte.

**Table 3.** Feature selection techniques used in this study.

Sl. no.	Feature selection method	Description	Features chosen	Feature names
1	Bat algorithm (BA) [26]	The algorithm mimics the behaviour of a bat (echolocation) to choose the best set of features	7	Monocyte, creatinine, potassium, D. bilirubin, ALT, ALP, protein
2	Cuckoo search algorithm (CSA) [27]	The algorithm imitates the cuckoo bird’s nesting habits. Cuckoos use other birds’ nests as their own and lay their eggs there	7	Gender, haemoglobin, monocyte, basophil, ALT, ALP, HbA1c
3	Differential evolution (DE) [28]	It is a technique in evolutionary computation that improves a given problem by repeatedly attempting to enhance a potential solution	5	Monocyte, eosinophil, ALT, ALP, HbA1c
4	Firefly algorithm (FA) [29]	The algorithm mimics the flashing behaviour of fireflies to choose the best set of features	6	Haemoglobin, creatinine, potassium, AST, ALT, ALP
5	Flower pollination algorithm (FPA) [30]	The algorithm is based on the concept of pollination in flowers	6	Haematocrit, eosinophil, basophil, T. bilirubin, AST, ALP
6	Genetic algorithm (GA) [31]	The technique uses the theory of natural selection to improve the solution (set of features) after each iteration	4	Haemoglobin, eosinophil, ALT, HbA1c
7	Grey wolf optimization (GWO) [32]	The algorithm is based on the pack behaviour of wolves. The solutions are divided into alpha, beta, gamma and omega. The best solution (set of features) is denoted using alpha	4	Haemoglobin, AST, ALT, ALP
8	Harris Hawks optimization (HHO) [33]	The algorithm mimics the hunting technique of Harris Hawk birds	3	Monocyte, AST, ALP
9	Jaya algorithm (JA) [34]	A population-based algorithm underlies it. It incrementally raises the quality of the population’s proposed solutions	7	Gender, TWBC, basophil, sodium, T. bilirubin, AST, ALP
10	Particle swarm optimization (PSO) [35]	The algorithm mimics the behaviour of a flock of birds/school of fish. The candidate solution is improved in each iteration using parameters such as velocity and position	5	Haemoglobin, haematocrit, AST, ALP, HbA1c
11	Salp swarm optimization (SS0) [36]	The algorithm mimics the formation of sea salps to choose the best features	7	Gender, haemoglobin, NLR, basophil, potassium, AST, ALP
12	Sine cosine algorithm (SCA) [37]	Mathematical models such as sine and cosine functions are used to choose the optimal number of features	3	Gender, D. bilirubin, ALT
13	Whale optimization (WO) [38]	Foraging behaviour of hump back whales (bubble nest method) is used to improve the candidate solution and choose the best features	2	Basophil, HbA1c
14	Mutual information (MI) [39]	The algorithm is based on the concept of information gain and entropy	10	HbA1c, sodium, potassium, urea, T. bilirubin, ALP, ALT, creatinine, basophil and AST
15	Pearson’s correlation (PC) [40]	Pearson’s correlation coefficient (*r*) is used to identify the importance of each feature. The value of *r* lies between −1 and 1	10	Eosinophil, TWBC, albumin, gender, platelet, sodium, bilirubin, ALP, HbA1c, bilirubin

### 2.3. Machine learning pipeline and performance metrics

Several ML algorithms were used for model training. The ideal hyperparameters for each model were found using the grid search method [41]. A fivefold cross-validation approach was additionally applied during training. It separates the data into several subgroups for training and testing [42]. The models become more reliable, when the data are divided into folds. Further, all the models were stacked on various levels.

**Figure 5.**
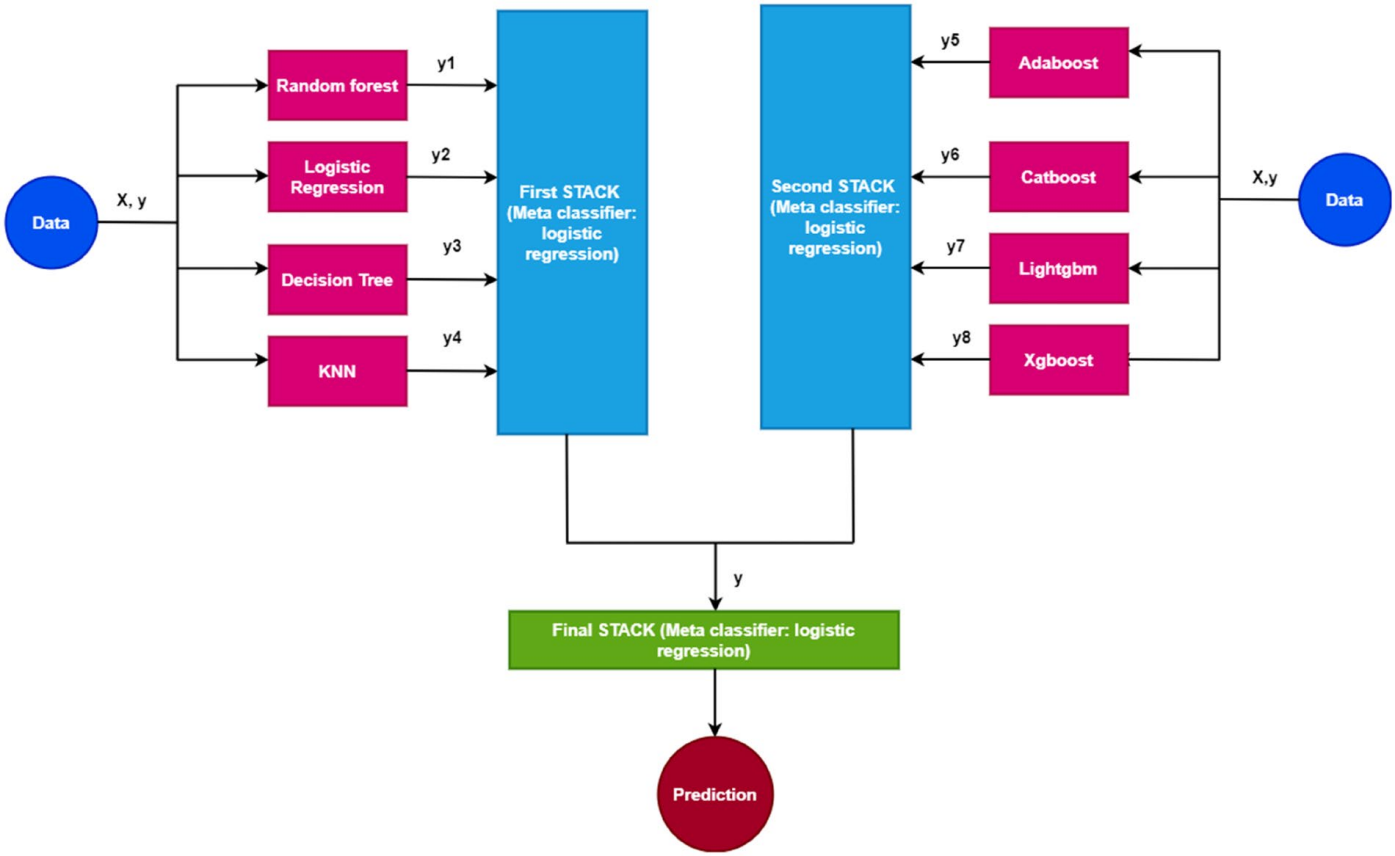
Custom stacking architecture to predict COVID-19 diagnosis.

Stacking is a method of collective learning that integrates the results of several classifiers using a meta-learner. The meta-learners maximize each model’s abilities while minimizing the weakness of the associated baseline classifiers. This custom stacking technique results in a better and more reliable classifier [43]. The first stacked model was formed using logistic regression, decision tree, K-nearest neighbors (KNN) and random forest. The boosting classifiers such as adaptive boosting (AdaBoost), extreme gradient boosting (Xgboost), categorical boosting (Catboost) and light gradient boosting machine (Lighgbm) were aggregated to form the second stack. The final stack was ensembled using the first and second stacks. The final stacked model can be used for prediction since it combines several heterogeneous classifiers. All stacking models used logistic regression as their meta-classifier. The customized stacking architecture is pictorially represented in Figure 5.

Further, DL algorithms were tested. Deep learning is a part of ML, which processes datasets according to a predetermined mathematical framework to discover patterns and relationships [44]. While ML needs feature selection, DL automatically performs feature mining and modelling steps during training. It can also generate new features, handle unstructured data, perform self-learning tasks and support parallel processing.

Complex irregular patterns can be modelled by DNNs [45]. Its main job is to manage user inputs, run increasingly complex calculations on the data, and generate outcomes that can aid in decision-making.

CNN was primarily used for imaging data. Tabular (one-dimensional) data can also make use of CNN [46]. A benefit of 1D-CNN is that it allows users to derive insights from the data without needing specialized knowledge.

Long short-term memory networks is another DL algorithm which uses a sequence of neural networks and allows information to be stored for a long period [47]. It solves the vanishing gradient problem that recurrent neural networks (RNN) commonly face. Machine translation and natural language processing applications make use of LSTM. However, it can be used for time-series data. LSTM contains three gates: Forget gate, Input gate and Output gate. LSTM comprises a memory cell (LSTM cell), which acts like a feed-forward neural network. Further, each neuron has a current state and a hidden layer. The Forget gate decides to keep the information or delete it. The Input gate processes new/updating of information. The output gate passes the information to the next layer.

ResMLP is another efficient model used in DL. It was developed by Touvron et al. [48]. ResMLP is based on the principle of multi-layer perceptron’s (MLP).

**Figure 6.**
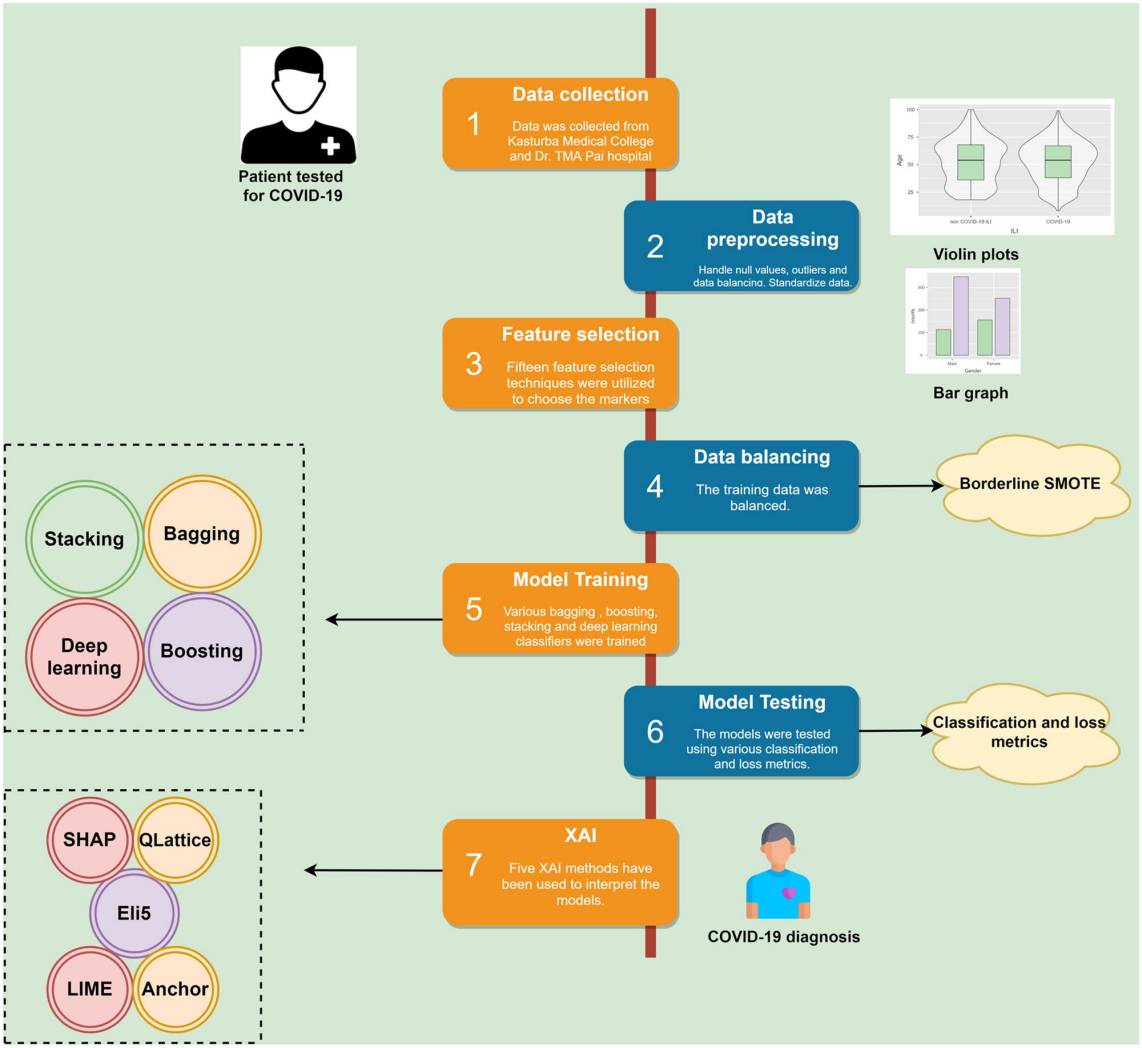
Machine learning pipeline for COVID-19 diagnosis.

A collection of frameworks and tools called XAI are designed to comprehend and analyse the models/classifiers [11]. SHAP, LIME, Eli5 and QLattice were the explainers utilized in this study. The entire flow diagram of the ML pipeline is depicted in Figure 6. Table 4 describes the performance metrics used to validate the classifiers. Several classification and loss metrics have been considered to evaluate the classifiers.

**Table 4.** Performance metrics used in this study.

Sl. no.	Metric name	Formula	Description
1	Accuracy	$\mathrm{Accuracy}=\frac{\mathrm{TP}+\mathrm{TN}}{\mathrm{TP}+\mathrm{TN}+\mathrm{FP}+\mathrm{FN}}$ (1)	It measures how many correct predictions a machine learning model makes.
2	Precision	$\mathrm{Precision}=\frac{\mathrm{TP}}{\mathrm{TP}+\mathrm{FN}}$ (2)	It measures the number of positive classifications, which were actually correct. The precision is high when false positive results are low
3	Recall	$\mathrm{Recall}=\frac{\mathrm{TP}}{\mathrm{TP}+\mathrm{FN}}$ (3)	It gauges how well the model can identify the positive class. The recall is high when false negative results are low.
4	*F*1-score	$F1\text{-}\mathrm{score}=\frac{2\times \mathrm{Precision}\times \mathrm{Recall}}{\mathrm{Precision}+\mathrm{Recall}}$ (4)	It is a metric, which combines both precision and recall.
5	AUC	–	In receiver operating characteristic curve (ROC), true positive rate is plotted against false positive rate at various thresholds. The area under this curve is called AUC (area under curve).
6	Average precision (AP)	–	In precision–recall curve, precision is plotted against recall at various thresholds. The area under this curve is called average precision.
7	Jaccard score (JS)	$\text{Jaccard score}=J\;(A,B)=\;\frac{\left|A\cap B\right|}{\left|A\cup B\right|}$ (5) where *A* and *B* are the two classes	The degree of similarity between two groups of data is gauged by the Jaccard score.
8	Log loss (LL)	$\text{Log loss}=-\frac{1}{N}\sum_{i=1}^{N}y_{i}\times \log\;\left(p(y_{i})\right)+(1-y_{i})\times \log(1-p(y_{i}))$ (6) where *N* is the number of samples.	How closely the prediction probability matches the true value is indicated by log loss
9	Mathew’s correlation coefficient (MCC)	$\mathrm{MCC}=\frac{\mathrm{TP}\times \mathrm{TN}\text{-}\mathrm{FP}\times \mathrm{FN}}{\sqrt{\left(\mathrm{TP}+\mathrm{FP})(\mathrm{TP}+\mathrm{FN})(\mathrm{TN}+\mathrm{FP})(\mathrm{TN}+\mathrm{FN}\right)}}$ (7)	It measures the difference between the actual value and predicted value.

## 3. Results and discussion

### 3.1. Model evaluation

Multiple models were used to diagnose mild–moderate COVID-19 patients. The grid search method of hyperparameter tuning was applied to each model. The data were further split using the fivefold cross-validation technique. To avoid overfitting, each model was trained 10 times, with the average results being computed.

The precision obtained by the classifiers for all feature selection algorithms is described in Table 5. The recall obtained is discussed in Table 6. Pearson’s correlation, mutual information and cuckoo search algorithm obtained excellent precision values among all the feature selection techniques. The final stacked model obtained an excellent precision of 84%, 81% and 86%, respectively. Feature selection algorithms such as differential evolution, genetic algorithm, particle swarm optimization and whale optimization obtained good precision values too. Grey wolf optimizer and since cosine algorithm obtained poor precision values with 66% and 65% for the final stacked algorithm. Excellent recall values were obtained by Pearson’s correlation, mutual information, cuckoo search algorithm, genetic algorithm, Jaya algorithm and particle swarm optimization. The final stacked model obtained a recall of 89%, 83%, 83%, 80%, 81% and 83%, respectively, for the above feature selection methods. The recall values obtained by the bat algorithm, firefly algorithm, grey wolf optimizer, Harris hawks’ method and sine cosine algorithm were comparatively poor.

**Table 5.** Precision obtained for various models for all feature selection techniques in percentage.

Machine learning methods	Feature selection techniques														
	BA	CSA	DE	FA	FPA	GA	GWO	HHO	JA	PSO	SSO	SCA	WO	MI	PC
Random forest	81	86	79	81	85	79	69	72	81	80	78	69	74	82	81
Logistic regression	50	57	63	52	71	65	48	61	64	61	56	52	61	58	66
Decision tree	71	76	68	64	72	74	65	65	71	66	67	64	71	67	74
KNN	65	68	67	88	62	70	64	70	69	68	65	62	69	69	71
First STACK	68	74	71	67	69	71	65	71	70	69	65	65	75	73	76
AdaBoost	70	78	76	72	73	77	66	71	74	81	74	69	77	72	76
Catboost	73	83	78	73	80	80	65	70	74	80	76	66	74	80	81
Lightgbm	76	88	70	77	83	83	65	77	80	80	81	68	76	83	84
Xgboost	85	90	82	83	83	85	70	76	89	83	83	74	79	86	86
Second STACK	76	87	81	81	83	82	67	78	81	82	80	68	77	82	85
Final STACK	75	86	78	73	75	78	68	72	78	79	73	65	76	81	84

**Table 6.** Recall obtained for various models for all feature selection techniques in percentage.

Machine learning methods	Feature selection techniques														
	BA	CSA	DE	FA	FPA	GA	GWO	HHO	JA	PSO	SSO	SCA	WO	MI	PC
Random forest	76	87	82	74	82	78	73	74	83	84	78	75	77	82	85
Logistic regression	50	62	68	52	77	73	47	64	71	67	61	55	65	62	74
Decision tree	77	81	75	69	78	80	72	66	77	71	78	72	76	74	85
KNN	70	73	72	80	65	77	71	74	75	75	74	71	76	74	79
First STACK	73	77	75	70	72	78	71	75	76	75	74	73	78	77	86
AdaBoost	72	83	81	74	76	80	71	73	77	85	80	75	80	77	85
Catboost	75	85	82	74	81	83	73	73	81	87	82	74	77	85	87
Lightgbm	73	85	79	73	82	80	70	77	84	83	81	75	78	83	88
Xgboost	77	85	84	74	75	81	76	79	81	87	82	79	80	84	89
Second STACK	73	86	81	75	82	79	72	77	83	84	80	75	79	83	89
Final STACK	72	83	78	71	76	80	73	73	81	83	77	73	78	83	89

For further analysis, the best four feature selection techniques were chosen. Pearson’s correlation, mutual information, cuckoo search algorithm and particle swarm optimization were considered since all the above algorithms obtained good values for precision and recall. The highest precision and recall results among the four were obtained using Pearson’s correlation, with values of 84% and 89%, respectively. The results of the final stack were emphasized since they are a combination of many classifiers and are generally reliable. Seven other metrics were also computed and the results are summarized in Table 7. The final stack obtained a maximum accuracy of 89% when Pearson’s correlation and particle swarm optimization algorithms were utilized. A maximum *F*1-score of 86% was obtained by the final stack for Pearson’s correlation method. The *F*1-score obtained by the final stack for the cuckoo search algorithm, mutual information and particle swarm optimization was 84%, 82% and 81%, respectively.

**Table 7.** Performance of the ML classifiers after using the top four feature selection methods.

	Pearson’s correlation							Cuckoo search algorithm						
Algorithm/feature selection method	A	F1	AUC	AP	JS	LL	MCC	A	F1	AUC	AP	JS	LL	MCC
Random forest	90%	83%	0.93	0.93	0.89	3.28	0.64	90%	86%	0.92	0.98	0.88	3.43	0.66
Logistic regression	81%	70%	0.84	0.95	0.78	6.56	0.48	62%	59%	0.67	0.9	0.56	13.28	0.211
Decision tree	86%	80%	0.78	0.92	0.84	4.84	0.50	77%	78%	0.84	0.95	0.74	7.81	0.41
KNN	80%	75%	0.76	0.91	0.77	7.03	0.42	79%	70%	0.73	0.91	0.76	7.34	0.389
First STACK	84%	81%	0.90	0.98	0.81	5.62	0.49	81%	75%	0.87	0.97	0.78	6.72	0.41
AdaBoost	83%	80%	0.73	0.88	0.81	5.78	0.44	86%	80%	0.83	0.95	0.84	4.84	0.55
Catboost	90%	84%	0.93	0.98	0.88	3.43	0.66	86%	84%	0.89	0.97	0.84	4.84	0.56
Lightgbm	92%	86%	0.95	0.99	0.91	2.65	0.72	87%	86%	0.89	0.97	0.85	4.37	0.56
Xgboost	93%	87%	0.94	0.99	0.91	1.25	0.72	93%	86%	0.92	0.98	0.91	2.5	0.72
Second STACK	92%	87%	0.94	0.99	0.91	2.65	0.70	91%	86%	0.91	0.97	0.90	2.96	0.69
Final STACK	89%	86%	0.94	0.98	0.87	3.75	0.61	86%	84%	0.87	0.97	0.84	4.68	0.52
	Mutual information							Particle swarm optimization						
	A	F1	AUC	AP	JS	LL	MCC	A	F1	AUC	AP	JS	LL	MCC
Random forest	87%	82%	0.9	0.97	0.84	4.53	0.58	86%	82%	0.82	0.93	0.83	4.84	0.56
Logistic regression	65%	60%	0.55	0.84	0.61	12.12	0.16	66%	64%	0.65	0.88	0.63	11.12	0.07
Decision tree	79%	70%	0.8	0.93	0.75	7.34	0.44	81%	68%	0.78	0.90	0.78	6.56	0.47
KNN	77%	71%	0.75	0.91	0.73	7.97	0.41	80%	71%	0.74	0.89	0.76	7.03	0.43
First STACK	80%	75%	0.88	0.97	0.76	7.02	0.44	81%	72%	0.78	0.91	0.78	6.56	0.45
AdaBoost	86%	74%	0.84	0.94	0.84	4.68	0.57	79%	83%	0.78	0.9	0.76	7.18	0.43
Catboost	84%	83%	0.88	0.96	0.81	5.62	0.53	86%	84%	0.83	0.94	0.86	4.06	0.61
Lightgbm	87%	83%	0.9	0.97	0.85	4.37	0.60	88%	82%	0.82	0.93	0.86	4.06	0.63
Xgboost	92%	85%	0.92	0.98	0.91	2.65	0.72	88%	85%	0.83	0.94	0.85	4.21	0.61
Second STACK	88%	82%	0.91	0.97	0.85	4.21	0.61	88%	83%	0.87	0.93	0.85	4.21	0.64
Final STACK	88%	82%	0.90	0.97	0.86	4.06	0.62	89%	81%	0.81	0.97	0.86	4.22	0.63

A: accuracy; *F*1: *F*1 score; AP: average precision; JS: Jaccard score; MCC: Mathew’s correlation coefficient; LL: log loss.

**Figure 7.**
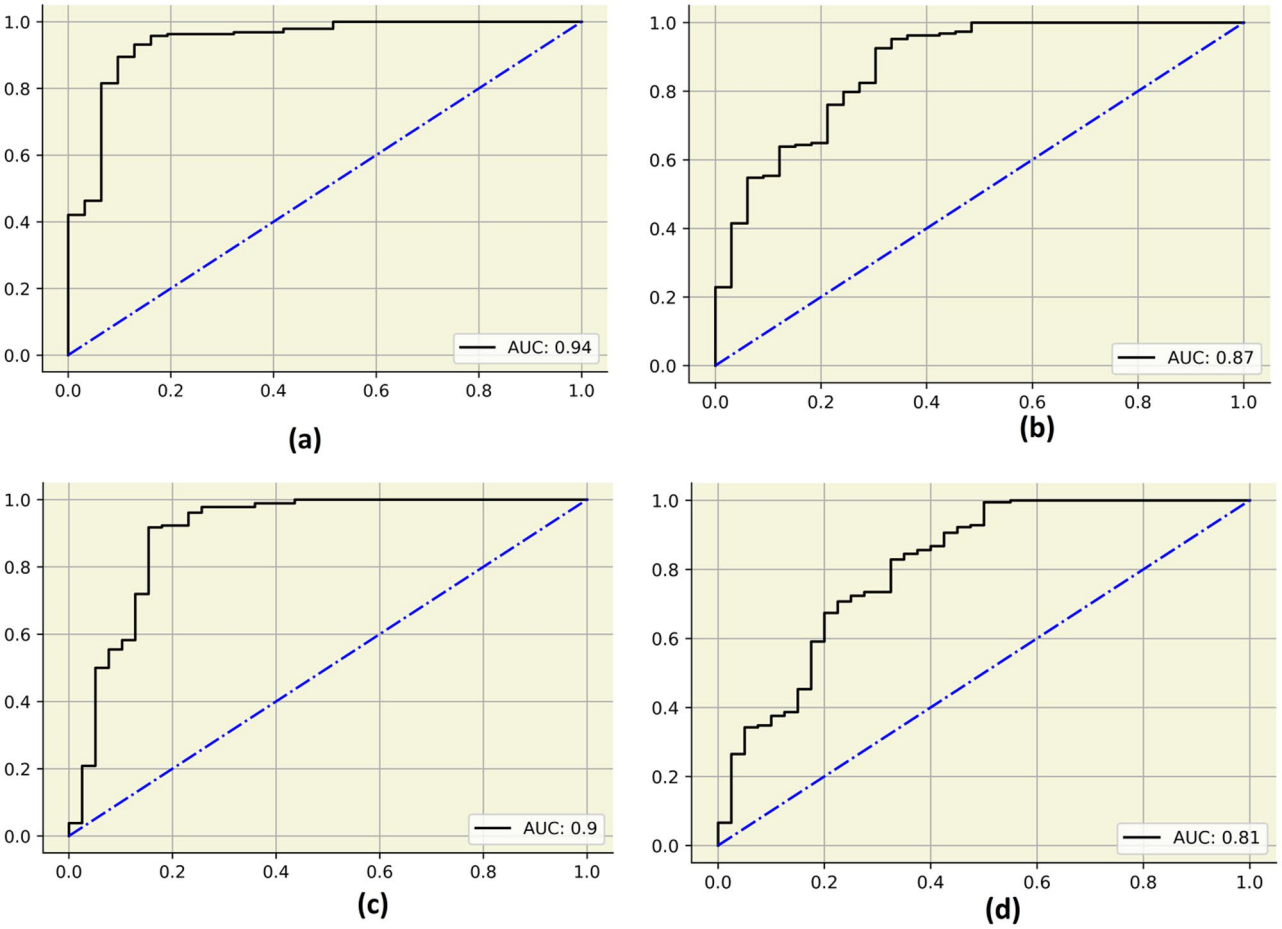
AUC curves obtained by the final stack for the test dataset. (a) Pearson’s correlation, (b) cuckoo search algorithm, (c) mutual information and (d) particle swarm optimization.

**Figure 8.**
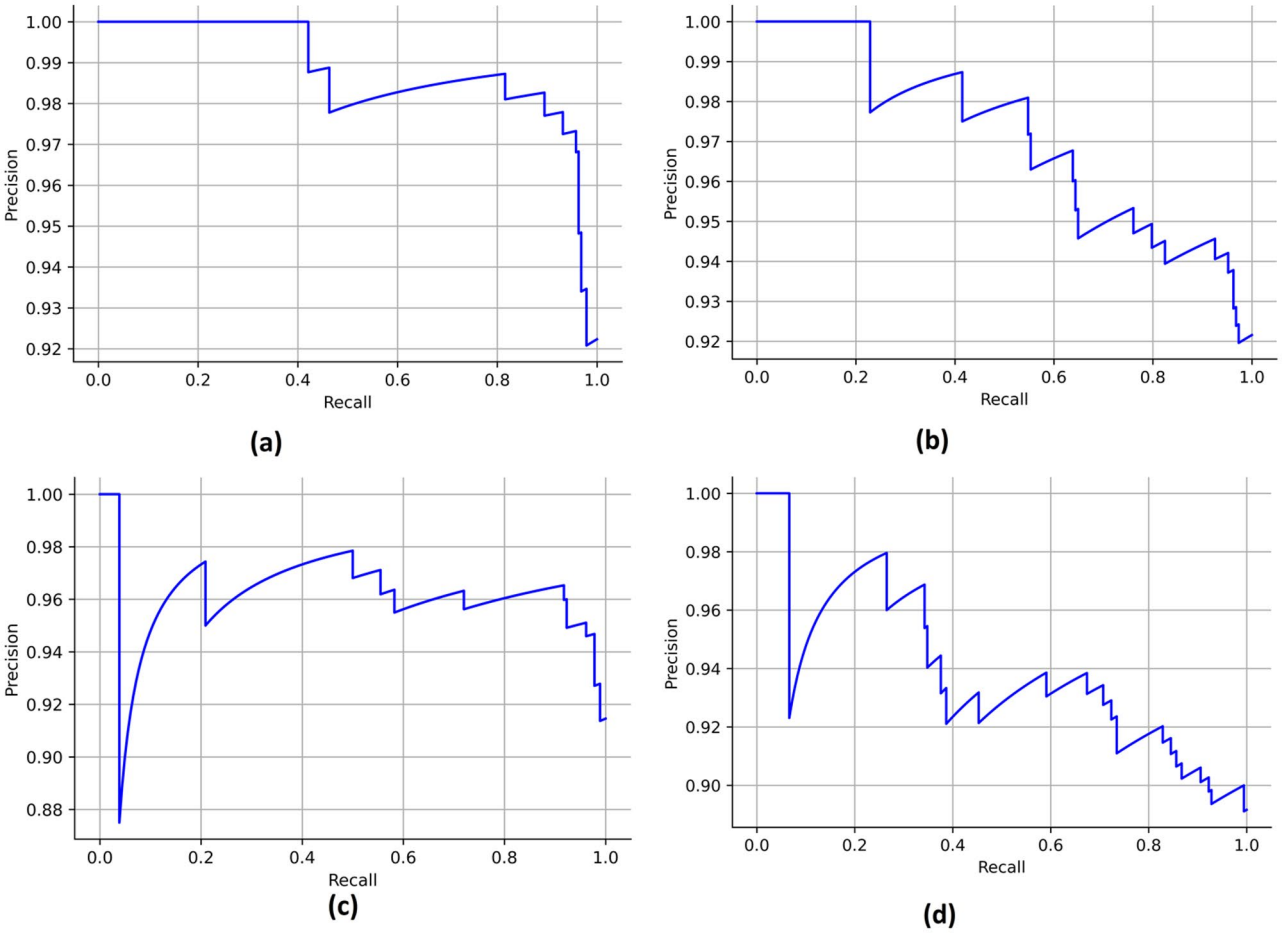
Precision–recall curves obtained by the final stack for the test dataset. (a) Pearson’s correlation, (b) cuckoo search algorithm, (c) mutual information and (d) particle swarm optimization.

A maximum AUC of 0.94 was obtained by the final stacked model for Pearson’s correlation technique. The AUC obtained by the final stacked classifier for the cuckoo search algorithm, mutual information and particle swarm optimization was 0.89, 0.90 and 0.84, respectively. The ROC curves for the final stacked models are described in Figure 7. Maximum average precision of 0.98 was obtained for Pearson’s correlation technique (final stacked model). The average precision obtained by the cuckoo search algorithm, mutual information and particle swarm optimization was 0.97, 0.97 and 0.97, respectively. The precision–recall curves are described in Figure 8. The final stacked model obtained a Jaccard score of 0.87 after using Pearson’s correlation technique. The log loss was minimum for Pearson’s correlation pipeline. The final stacked model obtained a log loss of 3.75. A maximum Mathew’s correlation coefficient of 0.03 was obtained for the particle swarm optimization pipeline (final stack).

**Figure 9.**
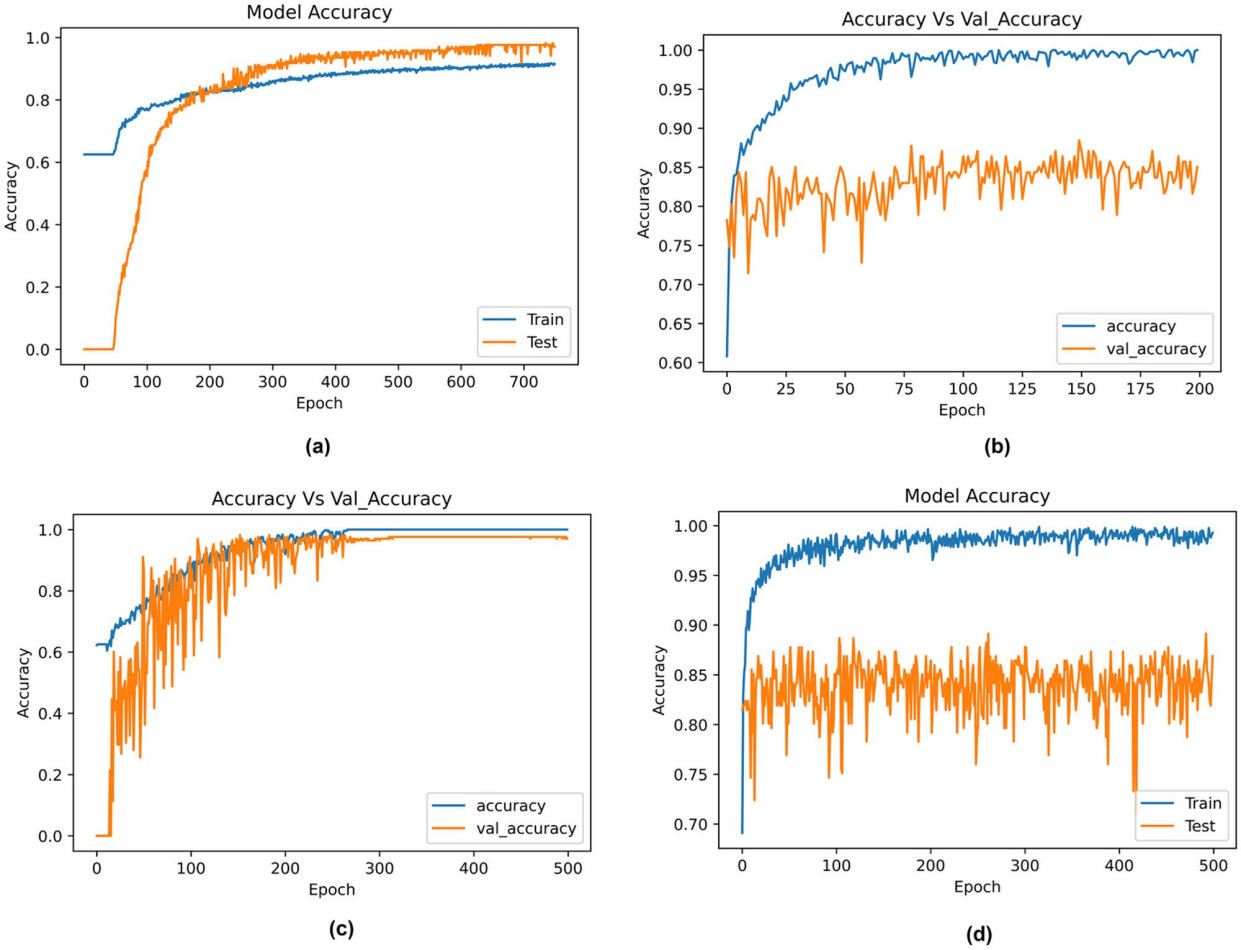
Accuracy curves obtained by the deep learning models. (a) DNN, (b) 1D-CNN, (c) LSTM and (d) ResMLP.

**Figure 10.**
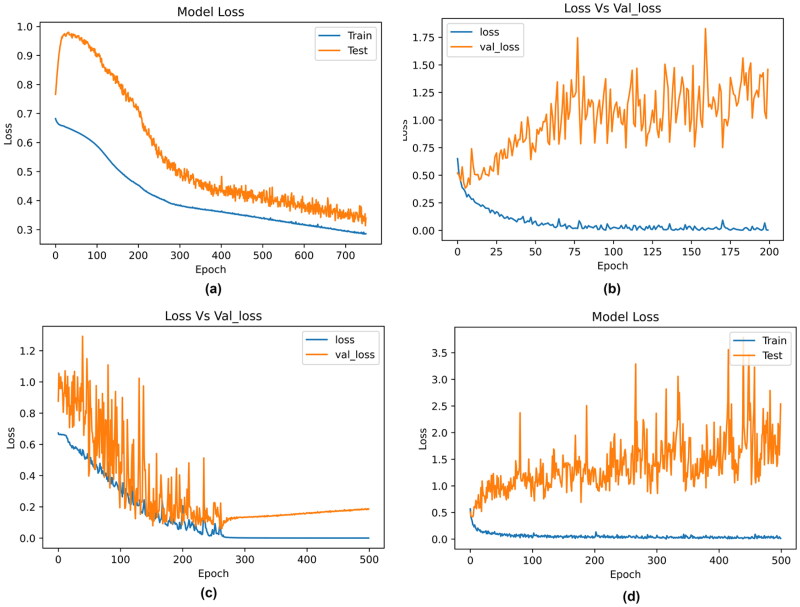
Loss curves obtained by the deep learning models. (a) DNN, (b) 1D-CNN, (c) LSTM and (d) ResMLP.

Further, the results obtained by the ML models were compared with DL algorithms. The model architectures of DNN, 1D-CNN, LSTM and ResMLP are described in Table 8. The results obtained by the neural network are described in Table 9. Among all the DL models, the 1D-CNN obtained the best results with an accuracy of 88%. The accuracy and loss curves for the classifiers are described in Figures 9 and 10. Here, the training accuracy is plotted against testing accuracy and training loss is plotted against validation loss. The 1D-CNN and ResMLP models were slightly overfitting compared to DNN and LSTM.

**Table 8.** Model architecture of the deep learning algorithms.

DNN			1D-CNN		
Model – sequential			Model – sequential		
Layer (type)	Output shape	Parameters	Layer (type)	Output shape	Parameters
dense (Dense)	(None, 24)	600	conv1d (Conv1D)	(None, 24, 32)	128
dense_1 (Dense)	(None, 11)	275	conv1d_1 (Conv1D) conv1d_2 (Conv1D)	(None, 24, 64) (None, 24, 128)	6208 24,704
dense_2 (Dense)	(None, 7)	84	max_pooling1d (MaxPooling1D)	(None, 12, 128)	0
dense_3 (Dense)	(None, 4)	32	dropout (Dropout)	(None, 12, 128)	0
dense_4 (Dense)	(None, 1)	5	flatten (Flatten)	(None, 1536)	0
Total parameters – 996 Total trainable parameters – 996 Total non-trainable parameters: 0			dense (Dense)	(None, 256)	393,472
			dense_1 (Dense)	(None, 512)	131,584
			dense_2 (Dense)	(None, 1)	513
			Total parameters − 556,609 Total trainable parameters − 556,609 Total non-trainable parameters: 0		
LSTM			ResMLP		
Model – sequential			Model – sequential		
Layer (type)	Output shape	Parameters	Layer (type)	Output shape	Parameters
lstm (LSTM)	(None, 24, 150)	91,200	dense (Dense)	multiple	800
lstm_1 (LSTM)	(None, 24, 75)	67,800	batch_normalization (Batch Normalization)	multiple	128
lstm_2 (LSTM)	(None, 50)	25,200	sequential (Sequential)	(None, 128)	107,776
dense (Dense)	(None, 1)	51	dense_17 (Dense)	multiple	129
Total parameters: 184,251 Total trainable parameters: 184,251 Total non-trainable parameters: 0			Total parameters: 108,833 Total trainable parameters: 108,769 Total non-trainable parameters: 64		

**Table 9.** Deep learning prediction results.

Model	Accuracy	Precision	Recall	*F*1-score	AUC	JS	LL	MCC	Hyperparameters
DNN	82%	73%	74%	73%	83%	0.79	6.25	0.96	Number of layers: 5, Neurons: {24,11,7,4,1}, Optimizer: Adam, Loss function: Binary cross entropy, Learning rate: 0.0001, Activation function: {Relu, Sigmoid for output layer}, batch size:10, epochs: 750
1D-CNN	88%	77%	79%	78%	85%	0.86	4.22	0.55	Number of layers: 9, Epochs:200, Batch size: 10, Optimizer: Adam, Loss function: Binary cross entropy, Learning rate: 0.001, Activation function: {Leaky relu, Sigmoid for output layer}
LSTM	84%	74%	73%	74%	84%	0.82	5.47	0.47	Number of layers: 4, Optimizer: Adam, Loss function: Binary cross entropy, Batch size: 32, Epochs: 500, Activation function: {Tanh, Sigmoid for output layer}
ResMLP	84%	72%	71%	71%	81%	0.82	5.47	0.42	Optimizer: Adam, Batch size:10, epochs: 500, Loss function: Binary cross entropy

The DL models performed well in classifying mild–moderate COVID-19. However, the stacked ML models were superior. When the data size is comparatively small, tree-based models can sometimes perform better than DL models for tabular data [49].

### 3.2. Explainable artificial intelligence to interpret model predictions

The classification system’s diagnosis will significantly influence how healthcare decisions are made. Technological advancements have led to digitizing and automating multiple processes and functions. As a result, accurate, intelligible and easy-to-understand techniques are given more priority. A comprehensible XAI model improves a healthcare professional’s capacity to validate the proposed predictions in the intricate world of medicine. Before making a final treatment choice, assessing the diagnostic model’s performance is essential. Furthermore, feature evaluations considering various factors are essential for resilient systems. In this research, five explainers have been utilized. They are SHAP, LIME, Eli5, QLattice and Anchor. Most of the algorithms were tested on the best four feature selection methods. When writing this manuscript, many explainers did not support DL models. Further, the ML pipelines obtained better results compared to DL models. Hence, DL models were not subjected to XAI techniques in this research.

**Figure 11.**
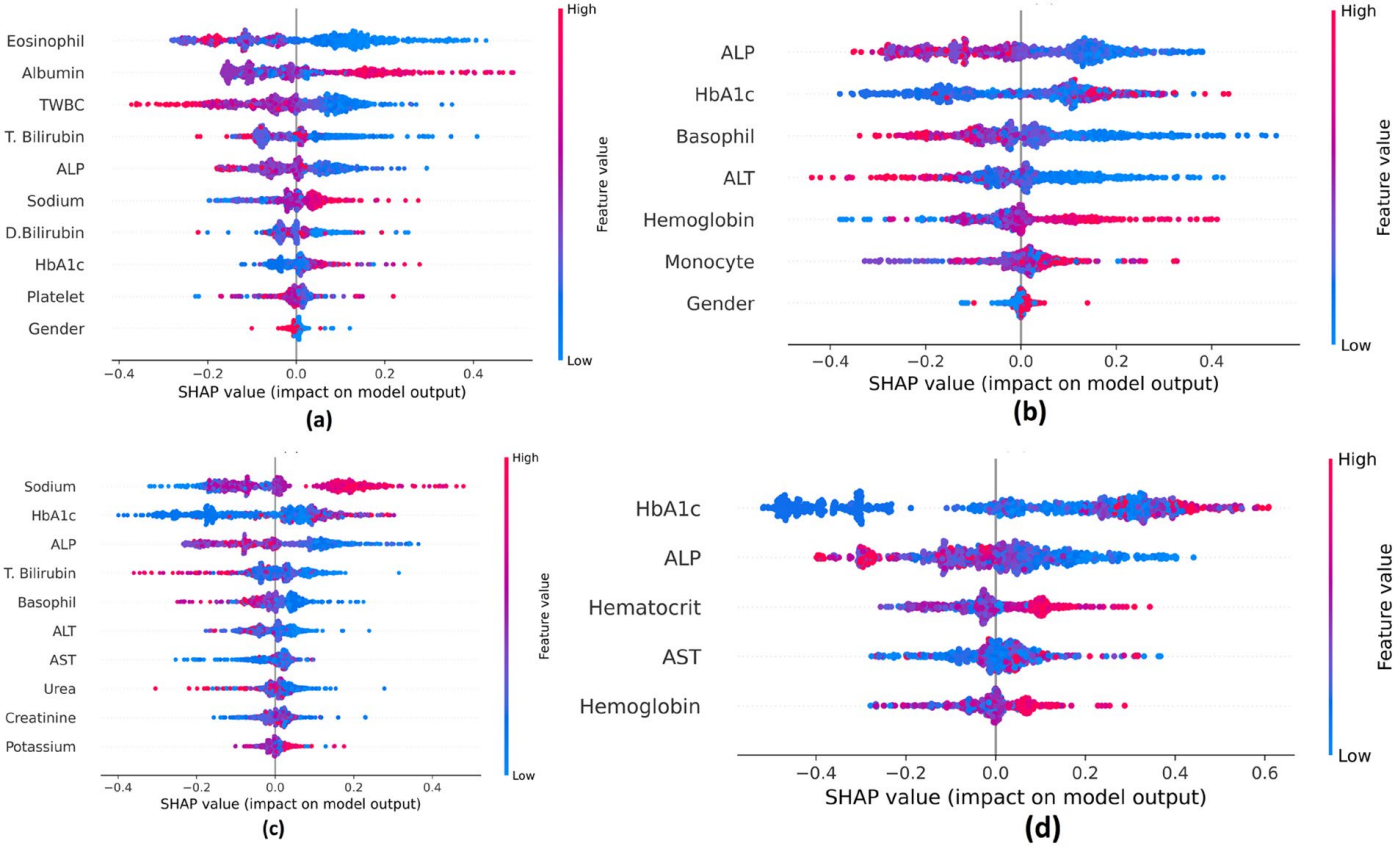
Beeswarm SHAP plots for the final stacked model. (a) Pearson’s correlation, (b) cuckoo search algorithm, (c) mutual information and (d) particle swarm optimization.

The basis of SHAP is probability and game theory [50]. The SHAP beeswarm plots for the final stack model for the best four feature selection techniques (PC, CSA, MI and PSO) are described in Figure 11. A hyperplane separates the two classes (non-COVID-19 ILI cases are towards the left, and COVID-19 positive cases are towards the right of the hyperplane). Additionally, the colours red and blue denote higher and lower values, respectively. Additionally, the markers are ordered in decreasing order of significance. (The best marker remains at the top). The figures show that markers such as eosinophil, albumin, TWBC, ALP, HbA1c, basophil and sodium are important. From the diagrams, it can also be seen that eosinophil count decreases in COVID-19 patients. Other markers including TWBC, ALP, ALT, basophil and T. bilirubin also decrease for SARS-CoV-2 patients. HbA1c was slightly elevated in COVID-19 patients. Other increased markers for COVID-19 patients are sodium, albumin, haemoglobin, haematocrit and monocytes.

**Figure 12.**
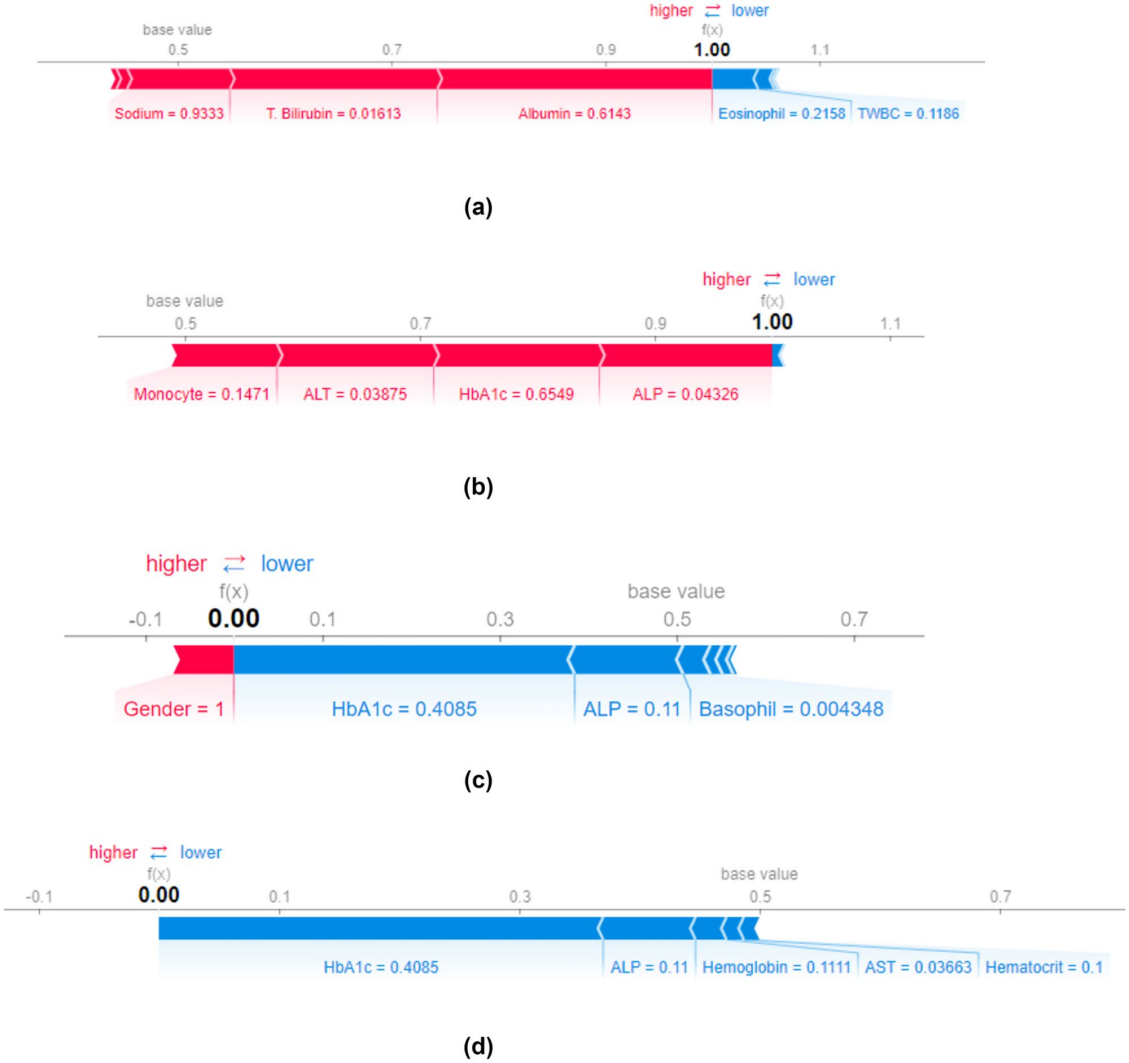
SHAP dependence plots for individual patient prediction. (a) Pearson’s correlation (COVID-19 positive), (b) cuckoo search algorithm (COVID-19 positive), (c) mutual information (COVID-19 positive) and (d) particle swarm optimization (COVID-19 negative).

A force plot can be utilized to predict an individual’s diagnosis. Figure 12 indicates force plots for individual patients for the best four feature selection methods. Figure 12(a,b) indicates a positive SARS-CoV-2 prediction. Figure 12(c,d) indicates a negative prediction. Markers such as albumin, T. bilirubin, HbA1c, ALP and ALT push the prediction towards COVID-19 positive. Clinical parameters such as HbA1c, ALP, haemoglobin and AST point towards a COVID-19-negative diagnosis. Further, the size of the bar is directly proportional to feature importance.

**Figure 13.**
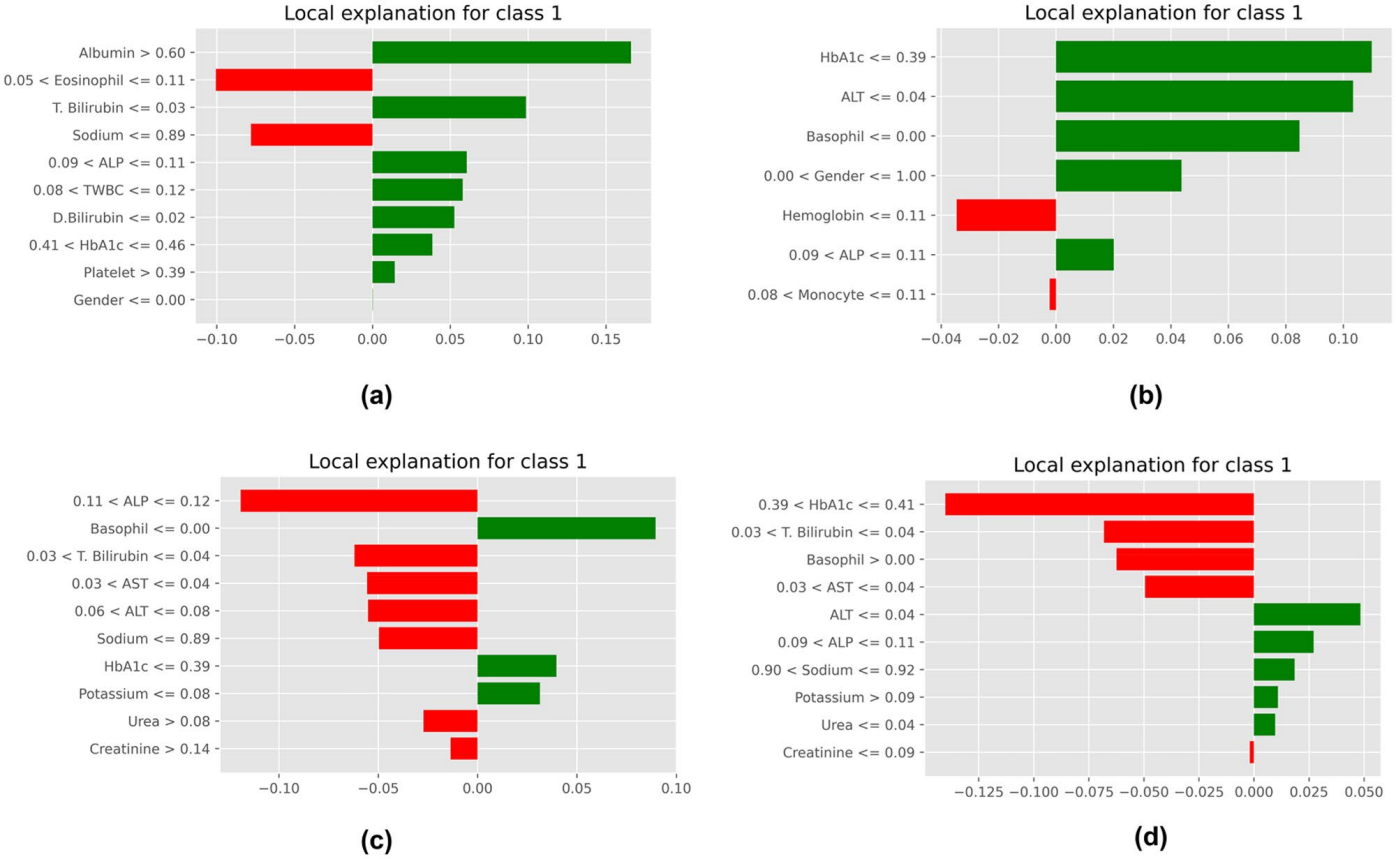
XAI using LIME. (a) Pearson’s correlation, (b) cuckoo search algorithm, (c) mutual information and (d) particle swarm optimization.

LIME is an XAI technique known for its local interpretations [51]. Initial data are updated to gain insight into the algorithm’s results after generating projections with the features chosen for explanations. It uses the linear regression technique to demystify the predictions. The algorithms generate many combinations, which are then used for training. Finally, justifications and analysis are offered for each prediction. The LIME explanations for the best four feature selection methods are depicted in Figure 13. Figure 13(a,b) depicts a COVID-19-positive patient. Every feature has a weight, and they are ranked in decreasing order of significance. As can be observed, a diagnosis for COVID-19 requires indicators like TWBC, ALP, T. bilirubin and eosinophil. Figure 13(b,d) shows that the COVID-19 test was negative. HbA1c, sodium and AST were crucial in predicting a COVID-19 negative diagnosis.

**Figure 14.**
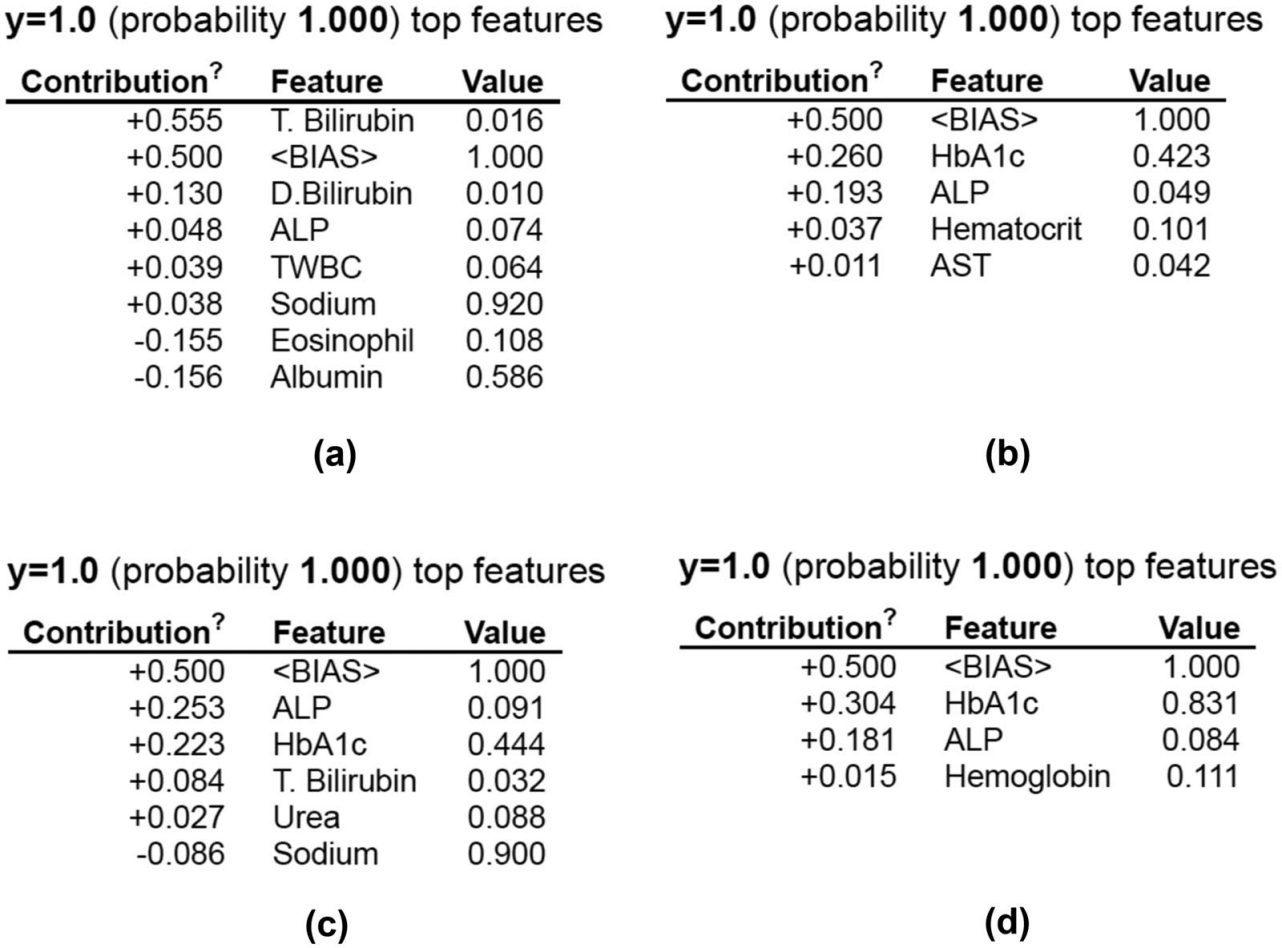
XAI using Eli5 for the decision tree model. (a) Pearson’s correlation, (b) cuckoo search algorithm, (c) mutual information and (d) particle swarm optimization.

A Python toolkit called Eli5 employs a standardized API to visualize and troubleshoot various classifiers [52]. It supports both regression and classification models. In this study, Eli5 was used on the decision tree models for the best four selection methods. Figure 14 depicts the explanations made by the Eli5 model. According to Eli5, the most critical parameters are T. bilirubin, D. bilirubin, HbA1c and ALP. The Eli5 also considers the ‘bias’ parameters for its predictions.

**Figure 15.**
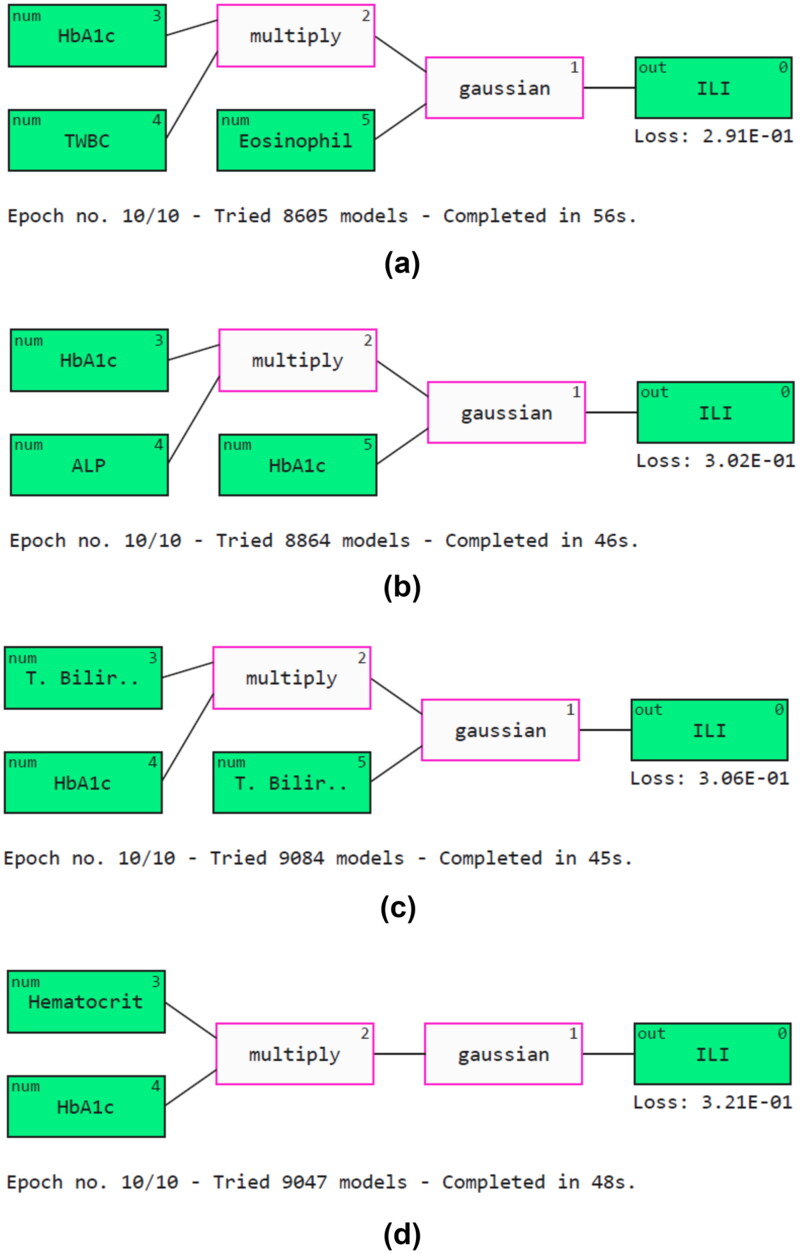
Model explainability using Qgraphs. (a) Pearson’s correlation, (b) cuckoo search algorithm, (c) mutual information and (d) particle swarm optimization.

An AI company, ‘Abzu’, developed QLattice. QLattice is a Python software module used for symbolic regression [53]. This explainer supports both categorical and numerical data formats. It enables users to quickly create, plot and examine essential features. Figure 15 illustrates how Qgraphs can be used to visualize the model’s interpretation. Qgraphs contain edges, nodes and activation functions. They also connect the input and output layers. In Python, Qgraphs are interpreted using the ‘FEYN’ library. The significance of indicators like HbA1c, TWBC, eosinophil, ALP and T. bilirubin can be observed. Further, the models have heavily used activation functions such as Gaussian and multiplication.

Anchor’ is another XAI method to interpret ML models [54]. Anchors use a set of ‘rules’ and ‘conditions’ to explain the important features. Each anchor (condition) is measured by two metrics: precision and coverage. Precision defines the accuracy of the explanations. The number of instances which use the same condition for prediction is defined by coverage. Anchor explanations are described in Table 10. The best markers that predict COVID-19 are: ALP, eosinophil, ALT, ALP and haemoglobin. The best markers that diagnose ILI non-COVID-19 patients are eosinophil, ALP, haematocrit, albumin and T. bilirubin.

**Table 10.** Anchor explanations for five COVID-19 positive patients and five COVID-19 negative patients for the best feature selection method (MI).

Instance	Patient prediction	Anchor condition	Precision	Coverage
1	ILI non-COVID-19	Eosinophil ≤ 0.01 AND ALP ≤ 0.09	0.95	0.13
2	ILI non-COVID-19	Eosinophil > 0.12 AND ALP > 0.11	0.74	0.15
3	ILI non-COVD-19	Haematocrit ≤ 0.10 AND Eosinophil > 0.07	0.79	0.13
4	ILI non-COVID-19	Albumin ≤ 0.56 AND Eosinophil > 0.07	0.81	0.35
5	ILI non-COVID-19	T. bilirubin > 0.03 AND Eosinophil > 0.07	0.79	0.36
6	ILI COVID-19	ALP > 0.09 AND Eosinophil > 0.07	0.80	0.44
7	ILI COVID-19	Haemoglobin ≤ 0.12 AND Eosinophil > 0.07	0.80	0.30
8	ILI COVID-19	Eosinophil ≤ 0.01 AND ALP ≤ 0.09	0.94	0.14
9	ILI COVID-19	0.06 < ALT ≤ 0.07 AND Eosinophil > 0.07	0.80	0.44
10	ILI COVID-19	Protein ≤ 0.80 AND Eosinophil > 0.07	0.79	0.40

### 3.3. Further discussions

This study identified clinical markers that can diagnose COVID-19 from similar respiratory diseases using ML algorithms. A combination of markers such as eosinophil, TWBC, T. bilirubin, HbA1c, ALP and ALT were crucial in diagnosing COVID-19. In order to validate the diagnosis, the decision support system can be used concurrently with an RT-PCR test.

Patients with COVID-19 had relatively decreased eosinophil levels. Eosinopenia in COVID-19 patients has already been documented in numerous researches [55,56]. HbA1c was comparatively higher in COVID-19 patients in this study. HbA1c has already been used as a prognostic marker in several COVID-19 studies [57,58]. Liver enzymes such as ALP, ALT, AST and T. bilirubin were comparatively lower in COVID-19 patients. Many studies have reported decreased liver enzymes among coronavirus patients [59,60]. According to our study, TWBC levels decreased in COVID-19 patients. In numerous researches, leukopenia was seen in COVID-19 patients [61,62]. Albumin levels were higher for COVID-19 patients in our study. According to other studies, higher albumin count was observed in mild–moderate COVID-19 patients [63,64]. Monocyte count tends to increase after contracting COVID-19 [65,66]. Our research agrees with the existing literature. Basophil count was lower in COVID-19 patients compared to ILI non-COVID-19 patients in our study. There are similar studies that report basopenia in COVID-19 patients [67,68].

Numerous research teams have developed AI systems intending to automate COVID-19 diagnosis in response to the unprecedented health crisis brought on by the global pandemic. However, very few studies have used clinical markers as a modality. Marateb et al. [69] used ML to distinguish between COVID-19 and non-COVID-19 pneumonia. The study considered three datasets and an ensemble classifier was utilized. All three datasets obtained good accuracies. White blood cells and C reactive protein were crucial for accurate diagnosis. Self-organizing maps, neural networks, and cluster algorithms were used to diagnose COVID-19 in another research [70]. The most critical markers were basophils, eosinophils, red cell distribution width and leukocytes. An XAI approach was used to diagnose COVID-19 in another research [71]. Four ensemble models were explained using LIME. The gradient-boosting decision tree obtained the best performance with an AUC of 0.86. The crucial markers were lactate dehydrogenase, white blood cells and eosinophil count. Guo et al. [72] used a multivariate algorithm to distinguish COVID-19 from similar respiratory diseases. A maximum accuracy of 92% was obtained, and the most essential markers were lymphocytes and white blood cells. Chadaga et al. [73] used ML and DL algorithms to diagnose COVID-19 from non-COVID-19 patients. A maximum accuracy of 96% was obtained by the final stack. The most crucial markers observed in this study were ALT, basophil and TWBC. However, severe COVID-19 patients were also included in the study. Another XAI technique was used by Rostami and Oussalah [74] to diagnose COVID-19. A graph-boosted XAI technique was used to interpret the results. The essential markers were platelets, eosinophils, C-reactive protein, AST and WBC.

In this study, we have used XAI techniques such as Eli5, QLattice and Anchor, which have been rarely used in medical research. COVID-19 severity has also reduced drastically after the introduction of COVID-19 vaccines. However, very few studies have included only mild and moderate COVID-19 patients. Therefore, we emphasize the suggested approach, which uses XAI to diagnose COVID-19 from other similar respiratory illnesses.

## 4. Limitations and future work

There are a few limitations to this study. Patients were considered from only one geographical location. It is necessary to consider patients from different demographics to establish reliable results. Medical validation was not performed. Real-time usage of models in hospitals can be performed. Graphical processing units (GPU) were not used in this study. Deep learning models run faster when GPUs are used. The data were imbalanced too. When the data are imbalanced, the models favour the majority class. To avoid ambiguity, the Borderline-SMOTE oversampling technique was used on the training dataset.

In the future, cloud-based systems can save the data and models. Unsupervised and reinforcement learning methods can also be utilized. Other diagnostic methods such as, cough sounds, CT scans and chest X-rays can also be combined suitably. Data from various countries and geographical territories can be combined to establish the results’ reliability. Hold-out set can also be considered along with training and testing/validation data. Oversampling techniques could also be compared with under sampling techniques.

## 5. Conclusions

In this research, several supervised learning algorithms were utilized to diagnose mild–moderate COVID-19 from other similar respiratory illnesses. The data were collected from two Indian hospitals. Before model training, 15 feature selection techniques were used to choose the most important parameters. A custom stacked model was then trained, which included several heterogeneous classifiers. Further, DNN, LSTM, 1D-CNN and ResMLP were also trained and tested. After using Pearson’s correlation and particle swarm optimization feature selection, the final stack obtained a maximum accuracy of 89%. Five XAI techniques, including LIME, SHAP, Eli5, QLattice and Anchor, were used to understand the predictions. The most important markers that were useful in COVID-19 diagnosis are eosinophil, albumin, T. bilirubin, ALP, ALT, AST, HbA1c and TWBC. The models can be deployed in real-time as a decision support system to validate the results obtained by the RT-PCR tests.

## Data Availability

Data will be made available on prior request to the corresponding author.
